# Low Temperature Vacuum Synthesis of Triangular CoO Nanocrystal/Graphene Nanosheets Composites with Enhanced Lithium Storage Capacity

**DOI:** 10.1038/srep10017

**Published:** 2015-05-11

**Authors:** Qun Guan, Jianli Cheng, Xiaodong Li, Bin Wang, Ling Huang, Fude Nie, Wei Ni

**Affiliations:** 1 Institute of Chemical Materials, China Academy of Engineering Physics, Mianyang 621900, Sichuan, P.R. China; 2 Sichuan R&D Center of New Materials, Mianyang 621900, Sichuan, P.R. China

## Abstract

CoO nanocrystal/graphene nanosheets (GNS) composites, consisting of a triangular CoO nanocrystal of 2~20 nm on the surface of GNS, are synthesized by a mild synthetic method. First, cobalt acetate tetrahydrate is recrystallized in the alcohol solution at a low temperature. Then, graphene oxide mixed with cobalt-precursor followed by high vacuum annealing to form the CoO nanocrystal/GNS composites. The CoO nanocrystal/GNS composites exhibit a high reversible capacity of 1481.9 m Ah g^−1^ after 30 cycles with a high Coulombic efficiency of over 96% when used as anode materials for lithium ion battery. The excellent electrochemical performances may be attributed to the special structure of the composites. The well-dispersed triangular CoO nanocrystal on the substrate of conductive graphene can not only have a shorter diffusion length for lithium ions, better stress accommodation capability during the charge-discharge processes and more accessible active sites for lithium-ion storage and electrolyte wetting, but also possess a good conductive network, which can significantly improve the whole electrochemical performance.

In recent years, transition metal oxides have been widely studied as promising anode materials for rechargeable lithium-ion batteries (LIBs), due to their abundance, low cost and high theoretical capacity^1^,^2^,^3^,^4^,^5^. Among various transition metal oxides, cobalt monoxide (CoO) has been considered as one of the most promising candidates owing to its high theoretical Li-ion storage capacity^2^,^6^. Unfortunately, cobalt oxides suffer from poor capacity retention because of its low electrical conductivity, and large volume change during the charge-discharge processes^7^. To solve these problems, downsizing the cobalt oxides to nanoscale and constructing hybrid materials with these materials possessing highly conductive, and good mechanical properties, have been proven to be one of the most promising strategies^8^,^9^.

Graphene, a two-dimensional carbon atom monolayer, can serve as conductive matrixs and stress buffering layer to improve lithium-ion storage performance. So far, a number of hybrid materials consisting of graphene and metal oxides, such as Fe_3_O_4_/Fe_2_O_3_^10^,^11^, SnO_2_^12^,^13^,^14^, Co_3_O_4_/CoO^1^,^9^,^15^,^16^,^17^, Mn_3_O_4_^18^,^19^, V_2_O_5_^20^,^21^, TiO_2_^22^,^23^,^24^, have been reported as anode materials for LIBs and exhibit good electrochemical performance with high capacity and excellent rate capability. It has been well known that the dimensional size and dispersion uniformity of the oxide on the GNS are vital to the electrochemical performance of the electrode. Further improvements of cell performance can be achieved by using the oxide with smaller particle size and more uniformly dispersing on the GNS^3^,^6^,^25^,^26^. Because small nanosized particles with good dispersion on the GNS can provide a large specific surface area to buffer the volume change of metal oxides during the charge/discharge processes, shorten the diffusion length for lithium-ion, and facilitate fast electrons transport. These characteristics can lead to excellent capacity retention and good cycling performance. However, most of metal oxide/GNS composites have a relatively large and random dispersion oxide on the GNS^6^,^8^,^26^. Meanwhile, the in-situ chemical synthetic procedures commonly used for the metal oxide/GNS composites are carried out with complicated processes accompanying with long time and high-pressure (hydrothermal or solvothermal method)^23^,^24^,^27^. Furthermore, the reaction process generally involves strong reduction reagents such as hydrazine, sodium borohydride and different surfactants due to relatively poor manipulation on metal oxide/GNS^23^,^28^,^29^. Zhang *et al.*^30^ synthesized the nanoparticle CoO/GNS composites by a facile in situ synthesis method. Though the Coulombic efficiency is 95%, an initial charge capacity of CoO/GNS composites was only 735.7 mAh g^−1^. Zhang *et al.*^31^ prepared CoO/GNS/C composites by electrospinning with the poisonous DMF as the solvent. However, the discharge capacity was only 1036 mAh g^−1^ at a current density of 100 mA g^−1^ for about 40 cycles. Besides, the performances of similar reported works of CoO/carbonaceous materials were also summarized in Table SI1. So it is highly desired to develop a mild method to synthesize metal oxide/GNS composites with smaller nanoparticle sizes and better-dispersion for high-performance LIBs.

In this paper, we design a mild method to synthesize triangular CoO nanocrystal/graphene nanosheets composites by low temperature processes (see Fig. 1). First, the cubelike Co_5_(OH)_2_(CH_3_COO)_8_•2H_2_O precursor is obtained by a re-crystallization process of the ethanol solution of Co(CH_3_COO)_2_•4H_2_O at a low temperature of below −10 °C. Then the cobalt-precursor mixed with graphene oxide (GO) to form the mixture of the cubelike Co_5_(OH)_2_(CH_3_COO)_8_•2H_2_O and GO (step I). Second, the CoO nanocrystal/graphene nanosheets composites are obtained by low temperature high vacuum treating (step II), consisting of a triangular CoO nanocrystal of 2~20 nm well dispersed on the surface of GNS. When used as anode materials for lithium-ion batteries, the CoO nanocrystal/graphene nanosheets composites can deliver a high capacity of 1481.9 mAh g^−1^ after 30 cycles with a high Coulombic efficiency of over 96%.

## Results

**Figure 2:**
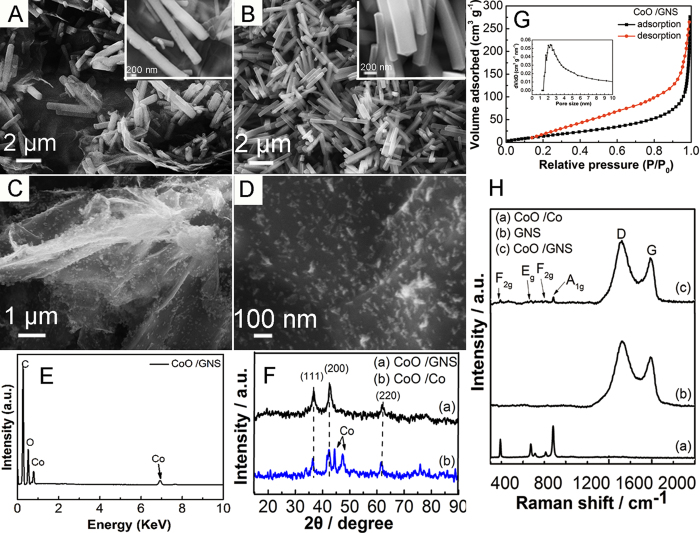
SEM images of (**A**) the prepared Co_5_(OH)_2_(CH_3_COO)_8_•2H_2_O-GO precursor, (**B**) the prepared Co_5_(OH)_2_(CH_3_COO)_8_•2H_2_O precursor, the inset is the high magnification image, (**C**) the nanocrystal CoO/GNS composites and (**D**) high magnification SEM of the nanocrystal CoO/GNS composites. (**E**) EDX spectrum of the nanocrystal CoO/GNS composites. (**F**) XRD patterns of the nanocrystal CoO/GNS composites (**a**) and CoO/Co (**b**). (**G**) Nitrogen adsorption-desorption isotherms of the nanocrystal CoO/GNS composites, and the inset is the pore size distribution curve. (**H**) Raman spectra of the CoO/Co (**a**), GNS (**b**) and nanocrystal CoO/GNS composites (**c**).

To reveal the reaction processes, different experiment conditions with and without the addition of graphene oxide were carried out. The morphologies of the CoO nanocrystal/GNS composites, CoO/Co composites and their precursors were studied by FESEM, respectively (Fig. 2A–D). It can be seen from the SEM image of Co_5_(OH)_2_(CH_3_COO)_8_•2H_2_O-GO precursor in Fig. 2A (XRD as shown in Figure SI1) that a large number of cubelike Co_5_(OH)_2_(CH_3_COO)_8_•2H_2_O are homogeneously distributed on the crumpled GO nanosheets. These cubelike nanorods are around 1~3 μm in length, 200 nm in width and 200 nm in height. The cross-section AFM image of GO (Figure SI2) suggests that the multilayered GO nanosheets are obtained with the thickness of 1.2~1.6 nm. While, without the addition of GO, the obtained precursors are cubelike Co_5_(OH)_2_(CH_3_COO)_8_•2H_2_O with similar size ranging from 2 μm to 5 μm (Fig. 2B). After thermal treatment under high vacuum, the cubelike nanorods in different precursors disappear and form the triangular nanocrystal CoO/GNS composites (Fig. 2C,D) and nanoparticle CoO/Co composites (Figure SI3). It can be seen clearly from the Fig. 2C,D that the CoO nanocrystals evenly dispersed on the surface of GNS, which may prevent the aggregation of nanocrystal CoO and GNS interlayer, thus benefiting the electrochemical performance of electrode. Without the addition of GO, only aggregated CoO/Co nanoparticles are obtained in the same synthesis processes. The high vacuum environment, accompanying the running out of reducing substance at a low temperature, brings an outward force on the acetoxy and hydroxyl group of the Co_5_(OH)_2_(CH_3_COO)_8_•2H_2_O and GO precursor, which helps to accelerate the decomposition of Co_5_(OH)_2_(CH_3_COO)_8_•2H_2_O-GO precursor, achieve a reduction of GO and form the cobalt oxide and GNS^25^. Without the addition of GO, the driving force under vacuum may take away the oxygen in the precursor and reduce the precursor to cobalt metal^25^. The reduction mechanism under high vacuum still need to be further investigated in the future. Fig. 2E shows an EDX mapping spectrum of the nanocrystal CoO/GNS composites. The strong peaks corresponding to C, Co and O elements can be attributed to the existence of graphene and nanocrystal CoO, respectively. Meanwhile, the elemental distribution of C, Co, and O in the nanocrystal CoO/GNS composites can be observed in Figure SI4, which demonstrates that the CoO nanocrystals are distributed uniformly on the GNS.

To further characterize the structure of the nanocrystal CoO/GNS composites and CoO/Co composites, the XRD tests were carried out (Fig. 2F). In the XRD patterns of nanocrystal CoO/GNS composites and CoO/Co composites, the sharp peaks at 36.5°, 42.4° and 61.5° can be attributed to the (111), (200), and (220) plane of cobalt mono-oxide (cubic CoO, JCPDS 01-089-7099), respectively. No obviously characteristic peak of GO at about 11° can be observed in the nanocrystal CoO/GNS composites, which suggests that the oxygen-containing groups of GO are removed at 300 °C for 10 min under high vacuum environment and the GO turns to GNS. Meanwhile, the characteristic (002) peak of GNS at about 25° is also disappeared, indicating that the GNS covered with well-crystallized CoO are obtained without obvious restacking and agglomeration of GNS^27^,^29^,^32^. In the XRD patterns of CoO/Co composites, besides the above diffraction peaks of CoO, two sharp peaks at 44.4° and 47.1° can be clearly seen, which can be attributed to metal cobalt. Moreover, the EDX spectrum of CoO/Co composites is also explored (Figure SI5). The molar ratio of Co/O is 1.17, which is larger than that of the pure CoO. It further confirms that the product contains certain amount of metallic cobalt, corresponding to the results of XRD test.

The pore properties of nanocrystal CoO/GNS composites and CoO/Co composites are further characterized by N_2_ adsorption-desorption isotherm at 77K in Fig. 2G and Figure SI6. BET specific surface area of the nanocrystal CoO/GNS composites and CoO/Co composites are 78.8 m^2^ g^−1^ and 70.9 m^2^ g^−1^, respectively. The Barret-Joyner-Halenda (BJH) pore size distribution (the inset of Fig. 2G) indicates that most pores are in the mesoporous range with a peak centered at approximately 2.5 nm. These pores can be formed from the gap of GNS interlayers and CoO nanocrystals. Fig. 2H presents the Raman spectra of the nanocrystal CoO/GNS composites, pure GNS and CoO/Co composites, respectively. For the nanocrystal CoO/GNS composites, four peaks below 1000 cm^−1^ can be attributed to the characteristic peaks of CoO. The peaks at 190 cm^−1^ and 595 cm^−1^ can be assigned to F_2g_ active mode of CoO, and the peaks at 465 cm^−1^ and 670 cm^−1^ can be attributed to the E_g_ and A_1g_ modes of CoO, respectively^33^. In addition, the disorder carbon (D band) at about 1350 cm^−1^ and graphitic carbon (G band) at about 1575 cm^−1^ are the characteristic peaks of carbonaceous materials, respectively. The intensity ratio (I_D_/I_G_) is a measure of disorder degree in the materials^34^,^35^. It can be known that the intensity ratio (I_D_/I_G_) of the pure GNS is 1.34. Compared with that of the pure GNS, the I_D_/I_G_ of nanocrystal CoO/GNS composites is 1.38, indicating the increased defects or edge areas from GNS to the nanocrystal CoO/GNS composites^33^.

**Figure 3:**
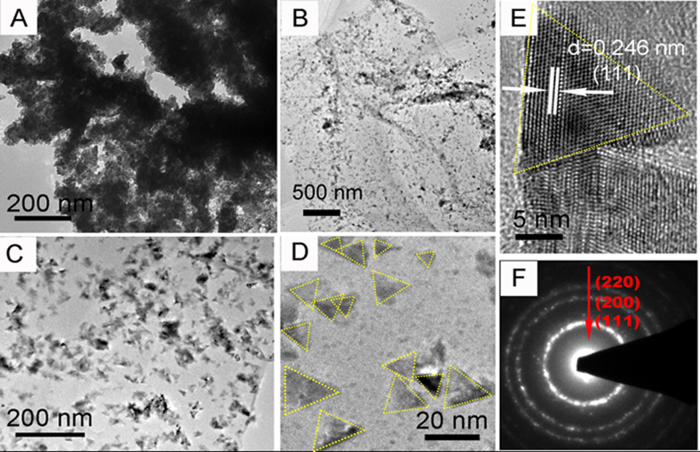
TEM images of CoO/Co (**A**) and nanocrystal CoO/GNS composites (**B**–**D**). HRTEM image of the nanocrystal CoO/GNS composites (**E**), and the electron diffraction pattern of the CoO circled (**F**).

The structures of the nanocrystal CoO/GNS composites and CoO/Co nanoparticle composites are further characterized by TEM and HRTEM (Fig. 3). After thermal treatment under vacuum, the cubelike Co_5_(OH)_2_(CH_3_COO)_8_•2H_2_O precursor is broken down and aggregated into irregular CoO/Co nanoparticles as shown in Fig. 3A. However, for the nanocrystal CoO/GNS composites, the TEM images (Fig. 3B–D) of an individual nanosheet exhibit a curled and rippled morphology consisting of a thin wrinkled paper-like GNS with evenly loading triangular nanocrystal CoO. Moreover, it can be seen that the size of triangular CoO nanocrystal is ranged from 2 nm to 20 nm, which is in good agreement with the SEM results. Besides, it also can be observed that a few smaller irregular shapes existed in the CoO nanocrystal/GNS composites, which may not wholly be assembled into the triangular nanocrystal. For the triangular nanocrystal CoO/GNS composites, the HRTEM image of a single CoO nanocrystal displays clear lattice fringes with a lattice spacing of about 0.246 nm, corresponding to the (211) plane of cubic CoO structure in Fig. 3E. In addition, the electron diffraction pattern demonstrates three diffraction rings corresponding to the (111), (200), and (220) plane of the face-centered cubic structure of CoO, respectively (Fig. 3F).

TG curves of the nanocrystal CoO/GNS composites and pure GNS displayed different weight loss processes (Figure SI7). For the pure GNS, a large weight loss occurs at 450~550 °C, which can be attributed to the oxidation of carbon skeleton^33^. Compared with pure GNS, the nanocrystal CoO/GNS composites show much lower thermal decomposition temperature. The main weight loss of the nanocrystal CoO/GNS composites occurs at 280~400 °C. These results indicate that the CoO existing in the composites could help to facilitate the decomposition processes^36^,^37^. The amount of GNS in the nanocrystal CoO/GNS composites is about 30.6 wt%.

**Figure 4:**
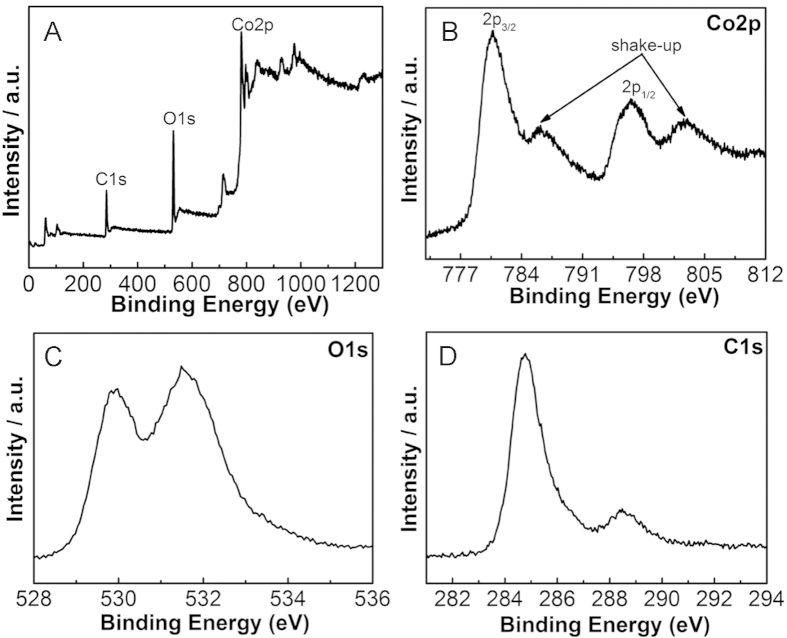
XPS spectra of the naocrystal CoO/GNS composites: (**A**) survey scan, (**B**) Co2p, (**C**) O1s, (**D**) C1s regions.

To determine the electronic state and the composition of the nanocrystal CoO/GNS composites, the XPS measurements were carried out. The XPS spectra of the nanocrystal CoO/GNS composites show three peaks at 285 eV, 531.3 eV and 781 eV, corresponding to the peaks of C1s, O1s and Co2p, respectively (Fig. 4A)^16^,^37^,^38^. The fine XPS spectra of Co 2p in Fig. 4B exhibit two peaks at 780.6 eV and 796.5 eV associated with two satellite peaks. The Co 2p_3/2_ peak at about 780.6 eV can be assigned to Co^2+^ coordinated to oxygen anion^39^. The satellite peak can be used as a fingerprint for the recognition of high spin Co (II) species in the CoO, originating from the occurrence of a ligand-to-metal charge transfer during the photoemission processes^40^. The spectra of the O1s region (Fig. 4C) show two peaks centered at 531.5 and 529.8 eV, correspond to the oxygen species in the CoO phase, and the OH species absorbed onto the surface of the composites, respectively. Moreover, the presence of the peak at 284.7 eV in the C1s spectra (Fig. 4D) can be ascribed to the graphitic carbon in GNS. However, the presence of peak at 288.7 eV in the C1s spectra can be assigned to the oxygen-containing groups in the composites^41^,^42^. The above results show that CoO is the main existence form of oxide on the surface of GNS.

## Discussion

**Figure 5:**
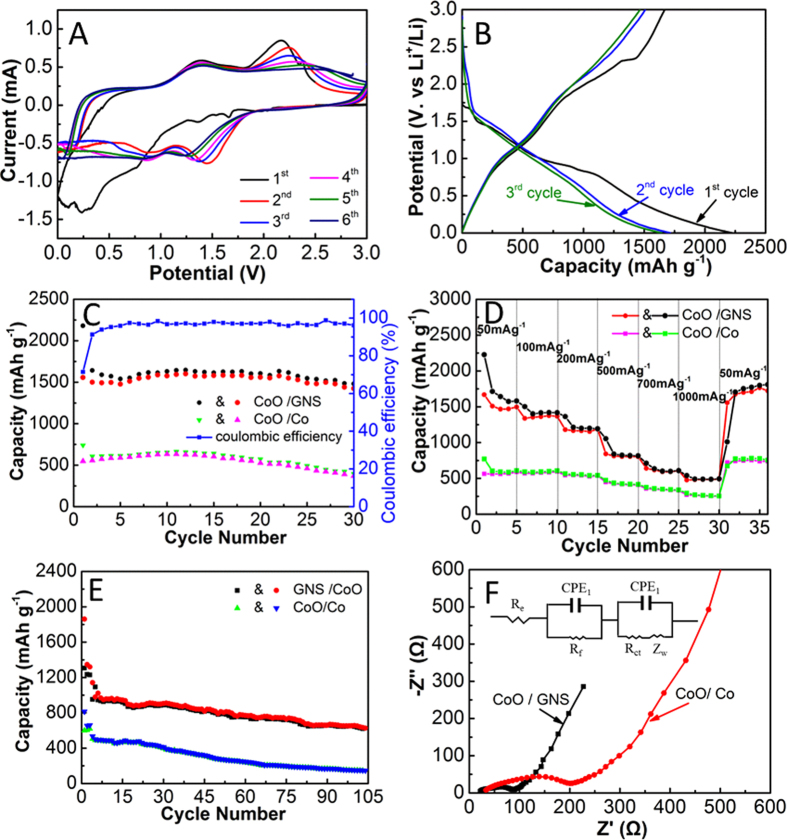
(**A**) Cyclic voltammograms of the nanocrystal CoO/GNS composites in a potential range of 0.02-3.0 V at a scan rate of 0.2 mV s^−1^. (**B**) Charge-discharge curves of the nanocrystal CoO/GNS composites at a current density of 100 mA g^−1^. (**C**) Cycling behaviors of the nanocrystal CoO/GNS composites and CoO/Co at a current density of 100mA g^−1^. (**D**) Rate performances of the nanocrystal CoO/GNS composites and CoO/Co at different current densities. (**E**) Cycling behaviors of the nanocrystal CoO/GNS composites and CoO/Co at a current density of 500 mA g^−1^. (**F**) Nyquist plots of the CoO/Co and the nanocrystal CoO/GNS composites after 50 cycles at a current density of 500 mA g^−1^, and the inset is an equivalent circuit model of the electrodes.

To investigate the mechanism of the electrochemical processes, the CV tests of the nanocrystal CoO/GNS composites at a scan rate of 0.2 mV s^−1^ within a voltage window of 0.02~3.0 V are shown in Fig. 5A. Two reduction peaks can be observed at about 1.36 V and 0.77 V in the first cycle of the nanocrystal CoO/GNS composites, which are ascribed to the insertion of Li^+^ into the CoO/GNS composites and the formation of a solid electrolyte interphase (SEI) film, respectively^29^,^43^. Two corresponding oxidation peaks are observed at about 1.3 V and 2.15 V. Furthermore, the reduction peak at 0.05 V and the broad oxidation peak at 0.27 V can be assigned to the insertion and extraction of Li-ion into/from the graphene, respectively. In the subsequent cycles, the reduction peaks shift to 0.85 V and 1.45 V and tend to be stable, which could be assigned to the formation of SEI film due to the decomposition of electrolyte by driving force of electrical field and the reduction of cobalt oxide to cobalt. Meanwhile, two broadened peaks in the oxidation process are shown at about 1.3 V and 2.2 V, respectively, corresponding to the partial decomposition of formed SEI and the reaction of Co and Li_2_O to form the CoO accompanying with Li^+^ extraction^44^.

Figure 5B shows the charge-discharge voltage profiles of the CoO/GNS composites at a current density of 100 mA g^−1^ in a voltage range of 0.02~3.0 V. The discharge and charge capacities in the first cycle are 2226 and 1669 mAh g^−1^, respectively, which are much higher than the theoretical CoO value of 716 mAh g^−1^. The extra lithium storage capability may be contributed from the enormous defects on the surface of the graphene, the reversible lithium-ion adsorption/desorption during the reversible SEI formation/decomposition processes and interfacial charge storage at the interface of different electrode components. The irreversible capacity loss could partly arise from the decomposition of electrolyte and the formation of the SEI layer in the first cycle. The subsequent cycles deliver close charge and discharge capacities. In accordance with the results of CV, two sloped potential plateaus at approximately 1.3 V and 0.8 V can be observed, corresponding to the reduction of CoO during the insertion of lithium ion and the formation of a SEI film, respectively. As indicated in Fig. 5C, the nanocrystal CoO/GNS composites show much higher reversible lithium storage capacity than CoO/Co composites. It can maintain a discharge capacity of 1481.9 mA h g^−1^ after 30 cycles at a current density of 100 mA g^−1^. Furthermore, the Coulombic efficiency rapidly increases from 71.4% in the first cycle to 96% in the fifth cycle and remains above 96% thereafter, which suggests facile conversion processes associated with efficient transport of ions and electrons in the electrodes. However, the CoO/Co composites can only retain the reversible capacity of 395.6 mA h g^−1^ after 30 cycles. These results suggest that the nanocrystal CoO/GNS composites can provide more active spaces, better stress accommodation capability and better diffusion pathway for lithium ions and electrons during cycling and hence leading to high capacity and excellent cycling performance.

To evaluate the electrode kinetics of CoO/Co composites and nanocrystal CoO/GNS composites, the rate capability was carried out as shown in Fig. 5D. It can be clearly seen that the nanocrystal CoO/GNS composites have a much higher specific capacity compared to the CoO/Co composites at the same conditions. The nanocrystal CoO/GNS composites still can keep a reversible capacity of as high as 609.1 mA h g^−1^ even charging-discharging at a higher current density of 1000 mA g^−1^. In contrast, the CoO/Co composites can only deliver a reversible capacity of about 220.8 mA h g^−1^ at the same current density. To further evaluate the cycling stability of the nanocrystal CoO/GNS composites and CoO/Co composites, the charge-discharge test at a constant current density of 500 mA g^−1^ is carried out (Fig. 5E). For electrode activation, all cells were cycled at a current density of 100 mA g^−1^ for the initial three cycles before cycling at a higher current density of 500 mA g^−1^. It can be observed that the initial discharge capacity of the nanocrystal CoO/GNS composites reaches to 1860.7 mA h g^−1^ at a current density of 100 mA g^−1^ in the first cycle, which is much higher than that of the CoO/Co composites (814.3 mA h g^−1^). When the current density increases to 500 mA g^−1^, the nanocrystal CoO/GNS composites have a discharge capacity of 1141.5 mA h g^−1^ and maintain 626.3 mA h g^−1^ after 104 cycles, indicating high cycling stability at higher current densities. However, the CoO/Co composites only retain a relatively low capacity of 146.5 mA h g^−1^. Furthermore, compare the capacity based on the active materials or total electrode as shown in Figure SI8, it can be clearly seen that the capacities of CoO/GNS are higher than that of CoO/Co, whether based on the active materials or the total electrode. Meanwhile, compared the electrochemical performance of the nanocrystal CoO/GNS composites with that of the previous reported results on CoO/carbonaceous materials, it can be observed from Table SI1 that the nanocrystal CoO/GNS composites have comparable or even superior performances in term of specific capacity and capacity retention.

The Nyquist impedance plots of the nanocrystal CoO/GNS composites and CoO/Co composites, acquired after charging-discharging 50 cycles, are shown in Fig. 5F. The semicircle at the high-frequency range can be attributed to the SEI film and/or contact resistance, the middle-frequency range semicircle represents charge-transfer impedance (Rct), and the inclined line at the low-frequency range corresponds to the lithium-ion diffusion processes (Warburg impedance)^45^,^46^. The results of fitting analysis indicate that the Rct values of the nanocystal CoO/GNS composites and CoO/Co composites are 30.8 Ω and 109 Ω, respectively. The Rct value of the nanocrystal CoO/GNS composites is much smaller than that of the CoO/Co composites. It demonstrates that the addition of GNS could increase the conductivity of the composites and decrease the charge-transfer impedance. In addition, the nanocrystal CoO/GNS composites show a more vertical Warburg line than CoO/Co composites electrode, indicating that the ion diffusion resistance in the nanocrystal CoO/GNS composites is smaller than that of CoO/Co composites in the electrochemical processes. These results further reveal that the nanocrystal CoO/GNS composites can offer good conductive network for fast Li^+^ diffusion, more accessible sites for Li^+^ storage and good structure stability for improved reversibility. All of these features would account for better electrochemical performance of the composites.

In conclusion, the nanocrystal CoO/GNS composites have been prepared by a mild low temperature synthesis route, consisting of a triangular CoO nanocrystal of 2~20 nm on the surface of GNS. First, cobalt acetate tetrahydrate is recrystallized at low temperature to form Co_5_(OH)_2_(CH_3_COO)_8_•2H_2_O with cubelike structure. Second, graphene oxide mixed with cubelike Co_5_(OH)_2_(CH_3_COO)_8_•2H_2_O to form a sandwich-like composites precursor. The CoO nanocrystal/GNS composites are obtained after annealing under high vacuum, which exhibit a high reversible capacity with a high Coulombic efficiency when used as anode materials for lithium ion batteries. The excellent performance of the nanocrystal CoO/GNS composites can be attributed to the good conductive network, more Li^+^ accessible sites and good structure stability.

## Methods

### Materials and reagents

All the chemical reagents used in this study are of analytical grade and used as received without further purification. All aqueous solution is prepared by deionized (DI) water.

### Synthesis of graphene oxide (GO)

In a typical synthesis, GO is prepared by a modified Hummers method. The detailed preparation process for GO could be found in our previous work^27^.

### Synthesis of CoO nanocrystal/GNS composites

First, 1.6 g of cobalt acetate tetrahydrate (Co(CH_3_COO)_2_•4H_2_O) was dissolved in 1000 mL of ethanol solution and kept at a temperature of below −10 °C for a few days. The precipitation was collected and dried in an oven at 60 °C for 8 h, and the cubelike Co_5_(OH)_2_(CH_3_COO)_8_•2H_2_O precursor was obtained^28^. Second, 0.1 g of GO powder was suspended in a 100 mL ethanol solution by ultrasonic treatment. The as-synthesized cubelike Co_5_(OH)_2_(CH_3_COO)_8_•2H_2_O precursor (0.3 g) was dispersed in a 100 mL ethanol solution and slowly dropped into the above GO ethanol solution under vigorous stirring. The mixture was kept at below −10 °C for one day. Then the composite precursor was separated by filtration and washed by ethanol. The as-prepared composite precursor was heated at 300 °C for 10 min under high vacuum environment (< 10 Pa). For comparison, GO and Co_5_(OH)_2_(CH_3_COO)_8_•2H_2_O were also treated at the similar procedure without the addition of Co_5_(OH)_2_(CH_3_COO)_8_•2H_2_O or GO, respectively.

### Materials characterization

The chemical composition of the samples was examined by X-ray diffraction (XRD, PANalytical, X’Pert PRO, Cu Ka). The morphology of the synthesized products was characterized using field emission scanning electron microscopy (FESEM, Carl Zeiss SMT Pte Ltd, Ultra 55) and atomic force microscopy (AFM, NSK, SPI3800N). Transmission electron microscopy (TEM) and high-resolution TEM (HRTEM) were carried out on a Libra 200 FE instrument at an acceleration voltage of 200 kV. Thermogravimetric (TG) analysis was carried out on a SDT Q600 instrument to determine the weight ratio of GNS to CoO. Raman spectroscopy was recorded from 100 to 3000 cm^−1^ on a Renishaw Invia Raman microscope excited by an argon ion laser beam. X-ray photoelectron spectra (XPS) were performed on Thermo Scientific Escalab 250 to analyze the surface chemistries of the samples. The N_2_ adsorption and desorption isotherm was obtained using a JW-BK300 apparatus.

### Electrochemical measurements

All working electrodes were fabricated by mixing active material, acetylene black (Super-P), and polyvinylidene fluoride (PVDF) binder with a weight ratio of 75 : 15 : 10 in N-methyl-pyrrolidone (NMP) to form a slurry on Cu foils current collector and then dried in a vacuum oven at 100 °C for overnight. The loading density of the active materials on the Cu foils is approximately 0.8 mg cm^−2^. The electrochemical properties of the electrode were evaluated using CR2032 coin-type cells assembled in an argon-filled glove box. Li metal foil was used as the counter and reference electrode. The electrolyte was 1 M solution of LiPF_6_ in ethylene carbonate (EC) and dimethylcarbonate (DMC) (1:1, v/v). The cells were charged and discharged galvanostatically between 0.02 V and 3.0 V using CT2001A battery test system (Land Co., Ltd.). The cyclic voltammetry (CV) tests and electrochemical impedance spectroscopy (EIS) measurements were carried out using a VSP (Bio-Logic SAS) electrochemical workstation. CV measurements of the electrode were performed in a range of 0.02–3.0 V at a scanning rate of 0.2 mV s^−1^. EIS testing was done with the frequency from 0.01 Hz to 1.0 MHz. The capacities of the electrodes are normalized by active materials and the total electrodes included active materials with graphene, acetylene black and PVDF.

## Author Contributions

B. W. and J. C. designed the experiments. Q. G. carried out the experiments. L. H. performed the XRD measurements. Q. G. and B. W. analyzed the datas and wrote the manuscript. J. C., X. L and W. N. contributed to the data analysis. B. W and F. N. provided the financial support. All the authors reviewed the manuscript.

## Additional Information

**How to cite this article**: Guan, Q. *et al*. Low Temperature Vacuum Synthesis of Triangular CoO Nanocrystal /Graphene Nanosheets Composites with Enhanced Lithium Storage Capacity. *Sci. Rep.* **5**, 10017; doi: 10.1038/srep10017 (2015).

## Figures and Tables

**Figure 1:**
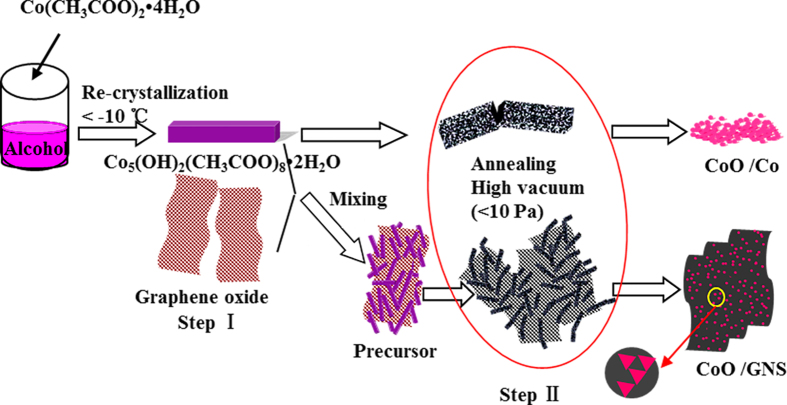
Schematic illustration for the low temperature formation processes of the nanocrystal CoO/GNS composites.
